# Histology-based Microstructural Tissue Phantoms for Realistic Ultrasound Simulation

**DOI:** 10.1177/01617346251406563

**Published:** 2025-12-29

**Authors:** Daniek A. C. van Aarle, Richard G. P. Lopata, Hans-Martin Schwab

**Affiliations:** 1 Photoacoustics and Ultrasound Laboratory Eindhoven (PULS/e), Department of Biomedical Engineering, Eindhoven University of Technology, Eindhoven, The Netherlands

**Keywords:** numerical tissue phantom, in silico modeling, ultrasound simulation, texture analysis, data generation

## Abstract

Ultrasound simulation has become an essential tool for transducer design, optimizing imaging strategies, and validating image analysis techniques. A simulation method that accommodates tissue-specific scattering would significantly improve realism of insilico phantoms, generating much needed training data with ground truth information (on anatomy, motion, function) available. This study presents a novel framework for constructing 2-D numerical tissue phantoms based on histological microstructure, enabling accurate and realistic ultrasound simulations. Whole-slide histology images of adipose fat, carotid artery, muscle, and skin were segmented to extract collagen and cellular components. Relative acoustic heterogeneity was estimated for all tissues, which was combined with the segmentations to generate spatial maps of density and speed of sound. Ultrasound simulations were performed using a pseudospectral wave solver and validated against ex vivo data. Quantitative analysis using the Jensen–Shannon Divergence and a multi-level texture anisotropy index demonstrated significantly improved realism in speckle patterns compared to baseline isotropic phantoms. The numerical phantoms combined with computed tomography-based patient geometries show promising results for realistic ultrasound dataset generation.

## Introduction

Medical ultrasound is often the non-invasive image modality of choice for medical diagnostics that require real-time imaging of soft tissues with high spatial and temporal resolution. Computer simulations have enabled the field to evolve, accelerating the development of new ultrasound transducers,^1^ improving image acquisition strategies, and enabling safety measurements and treatment planning.^2,3^ In addition, ultrasound simulation has been beneficial for the development and validation of image analysis techniques by providing ground truth for techniques such as image segmentation or strain imaging,^4-6^ in situations where the in vivo ground truth is inaccessible. One of the most commonly used simulation tools is Field II, which models linear ultrasound propagation using spatial impulse responses for a homogeneous medium.^7,8^ An approach similar to Field II can be found in SIMUS, a computationally efficient simulator in the frequency domain.^9^ Field II and SIMUS are well-suited for specific applications such as vector flow imaging and displacement tracking.^10-12^ However, these methods are less suitable for scenarios that need complex wave phenomena such as multiple scattering, diffraction and nonlinear effects, which play a significant role in soft tissue interactions. These limitations are overcome by numerical wave solvers, which enable accurate modeling of ultrasound wave propagation. Examples include finite-difference time-domain methods like Full-Wave^13^ and pseudospectral methods such as k-Wave.^14,15^

In order to achieve highly realistic ultrasound simulations, one needs a realistic numerical phantom besides a physically accurate wave solver. Currently, numerical phantoms employed in ultrasound simulation studies are typically based on anatomical structures extracted from computed tomography (CT) or simplified geometries.^16-20^ Here, the sub-wavelength organization of biological tissue cannot be resolved from CT. Usually this microstructure is then assumed to be isotropic, being modeled with a spatially random scatterer distribution. This assumption loses necessary detail in the resulting speckle patterns as actual intensity distributions are highly dependent on the underlying tissue type.

Previous work has shown that the inclusion of tissue microstructure, the spatial organization of tissue on a micrometer scale, directly derived from histological images, can substantially enhance the realism of simulated ultrasound data.^21-25^ Kraft et al. constructed a tissue phantom from a histology image of the coronary artery to simulate intravascular ultrasound (IVUS) data. Here, circular tissue-specific scatterers were assigned to manually segmented regions, yielding accurate speckle patterns compared to the measured IVUS.^23^ Bayat et al.^24^ simulated temporal-enhanced ultrasound using pathology-mimicking scatterers extracted from a histology slice, regarding cell nuclei as the main scattering source.^24^ On a larger length scale, in a recent study, histology images were used by Ostras et al.^25^ to model ultrasound B-lines originating in the superficial lungs.^25^

Although previous work shows the significance of including tissue microstructure, little emphasis is placed on anisotropic structures such as collagen fibers and cell shapes, losing necessary detail in the resulting speckle patterns. Qin et al.^26^ implemented myocardial fiber organization in their simulation approach, extracted from diffusion tensor imaging, enhancing the realism compared to an isotropic scattering approach.^26^ In addition to spatial distribution, local acoustic parameters, such as small changes in density and/or bulk modulus, strongly affect the tissue appearance. Previous work by Mamou et al.^21^ introduced 3-D impedance maps to identify scattering structures in tissue, relying on empirically assigning impedance values to histological structures by experts in the field.^21^ Later work employed impedance maps derived from acoustic microscopy to enhance Form Factor estimation.^27^ While these methods improve realism, they depend on expert annotation or acoustic microscopy, which limits scalability and accessibility. This research overcomes these limitations with an alternative solution to estimate the relative acoustic properties at a micrometer scale, to form a 2-D map of local density and speed of sound, without the need for absolute values. This enables simulations that account for refraction and aberration caused by local speed of sound differences, providing a practical method for realistic ultrasound simulation.

The aim of this research is to establish a method for the development of 2-D numerical tissue phantoms, based on tissue microstructure attained from histology samples, with the purpose of creating realistic and accurate ultrasound simulations. Unlike previous studies that consider histology to improve tissue simulation, we introduce a generic approach that models microstructure in terms of relative tissue heterogeneity, including the composition and orientation of cells and collagen fibers, extracted from whole- slide histology images. These tissue phantoms were resampled to a coarser simulation grid to reduce simulation time while maintaining the relevant microstructure. The numerical phantoms were imaged in silico using k-Wave,^14,15^ an acoustical simulator designed for time-domain simulations in complex media.

The method is evaluated on four different but often imaged tissue types, known to have a different appearance in US images, being: skeletal muscle, artery, adipose fat, and skin. The resulting images were analyzed quantitatively in terms of first-order speckle statistics, using the Jensen-Shannon Divergence, and compared to exvivo acquired ultrasound data of the same tissue sample with the same ultrasound transducers. To assess the structural appearance resulting from the underlying tissue, speckle anisotropy was quantified with a multi-level texture analysis. A comparison is made with numerical phantoms under the assumption that the tissue microstructure is totally isotropic. Finally, the added value of adding the microstructure is shown by combining the numerical tissue phantoms with computed tomography data to showcase an in vivo example of the carotid artery.

## Methods

**Figure 1.**
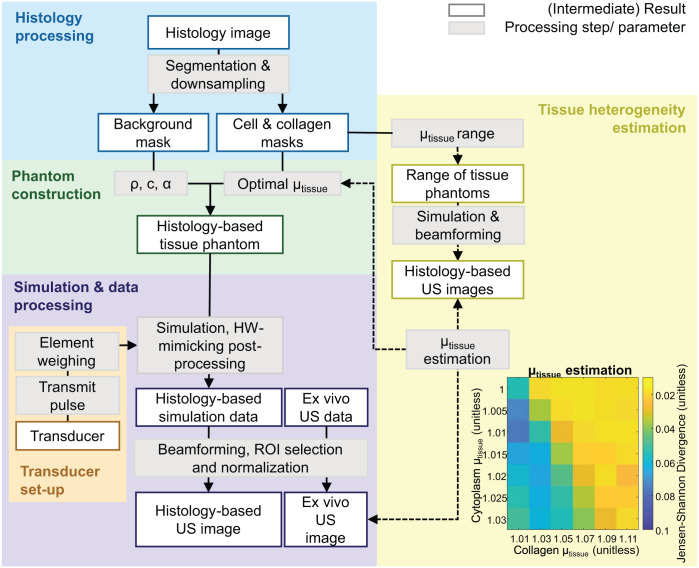
Overview of the simulation workflow, where histology-based tissue phantoms are constructed from histology images. The workflow is organized into five color-coded groups. In yellow, the method to estimate the relative tissue heterogeneity (μ_tissue_) is shown. An example of the μ_tissue_ parameter space is provided for the skeletal muscle, where the dissimilarity was computed between in silico and ex vivo ultrasound data using the Jensen-Shannon Divergence. Ex vivo ultrasound data was used to validate the method, undergoing the same beamforming and post-processing steps as the simulations. Abbreviations: density (ρ), speed of sound (c), acoustic attenuation (α), hardware (HW). *Note*. Please refer to the online version of the article to view this figure in color.

In this study, a framework for the development of numerical tissue phantoms is proposed. This numerical tissue phantom is a virtual, discretized representation of the ultrasonic imaging domain. It consists of a spatial map of the tissue density and speed of sound and is derived from segmented histology images. For each segmented tissue component, the optimal local acoustic properties were estimated by comparing in silico simulated ultrasound with ex vivo acquired ultrasound images of the same tissues. Figure 1 illustrates the complete simulation workflow, including the pipeline for creating the histology-based tissue phantom and estimating the relative acoustic heterogeneity (μ_tissue_). Ultrasound RF channel data were simulated in k-Wave, with a virtual representation of the transducer. The output in silico ultrasound images were compared to ex vivo ultrasound images in terms of first-order speckle statistics and texture analysis.

### Histology-Based Tissue Phantom

Porcine carotid artery, skeletal muscle (lower leg) and skin were obtained from a local slaughterhouse, originating from healthy pigs. Porcine fat samples were obtained from a local butcher, according to local regulations. Histological images of all biological tissues (carotid artery, adipose fat, muscle, and skin) were prepared. Tissue samples were placed in 4% formalin for complete fixation and were subsequently transferred into 15% and 30% sucrose in phosphate-buffered saline for cryopreservation. Samples were embedded in OCT compound and frozen using dry ice. Frozen sections were sliced with a thickness of 8 µm. The histology slides were stained with Weigert’s Iron Hematoxylin – Picrosirius Red staining. Picrosirius red stains collagen-rich tissue red, and cytoplasm and muscle fibers yellow. Weigert’s Iron Hematoxylin stains cell nuclei gray-brown. Whole-slide histology images were captured with a Leica DMi8 microscope (Leica Microsytems BV, Amsterdam, The Netherlands), at a magnification of 10× (0.32 NA) and a resolution of 0.43 µm/pixel.

The tissue microstructure was derived from the histology images by means of segmentation. As a pre-processing step, the histology images were transformed from RGB to the CIELAB colorspace. A *k*-means clustered image segmentation was used to decompose the tissue into two components: collagen and cells.^28^ Before constructing the tissue phantoms, the segmentations were post-processed using morphological opening and closing operations, to filter out small structures in the collagen masks. To ensure correct decomposition in two components, the images were segmented into multiple clusters which were later merged. The number of clusters and morphological operations were optimized for each tissue type. Further details can be found in Appendix A. The segmented collagen and cell components were low-pass filtered, to prevent aliasing during downsampling, and downsampled to the coarser simulation grid.^4^

The segmented collagen and cell components, representing the tissue microstructure, were superimposed on a tissue background to form the histology-based phantom. The background contained global acoustic properties (density, speed of sound, and absorption). The global acoustic parameters for each tissue type were chosen such that the average density and speed of sound of the resulting histology-based phantom matched the values derived from literature,^29,30^ an overview is listed in Table 1. Local acoustic variations were introduced by assigning relative changes in density and speed of sound to each of the microstructural components. These local acoustic parameters were defined in terms of tissue heterogeneity, *µ*_tissue_ (unitless), relative to the global properties. The power law acoustic absorption coefficient was set to 1.1 for all simulations. The skin phantom was modeled with a multilayered architecture, where the epidermis and dermis are modeled with the skin parameters, and the hypodermis (mainly subcutaneous fat) with the adipose fat parameters.

**Table 1.** Global acoustic properties for different tissues used in the microstructural tissue phantoms. The power law absorption coefficient (*y*) was assumed to be 1.1 for all tissues.

Tissue	Density [kg/m^3^]	Speed of sound [m/s]	Absorption [dB/(MHz^y^cm)]
Artery	1085	1565	0.6
Fat	911	1440	1.1*
Muscle	1085	1585	0.6
Skin	1100	1600	0.8
Water	1000	1480	2.2 *·*10^−3^

* Empirically derived.

The spatial density distribution of the histology-based phantom, *ρ*_tissue_ was defined as:

(1) $$\rho_{tissue}\left(x,z\right)=\rho_{tissue,global}\left(\begin{array}{l} M_{background}\left(x,z\right)\\ +\mu_{tissue}M_{microstructure}\left(x,z\right) \end{array}\right),$$

where *ρ*_tissue,global_ is the background density in kg/m^3^, *M*_background_ and *M*_microstructure_ represent binary spatial maps of the tissue background and microstructure components, respectively.

The local speed of sound varies with density according to a linear relationship originally introduced by Mast,^31^ and implemented in *k*-Wave:

(2) $$c_{tissue,local}=\left(\rho_{tissue,global}\mu_{tissue}+349\right)/0.893,$$

with *c*_tissue_ the speed of sound in m/s. The constants in this linear relationship are empirically derived based on literature values for soft tissues. The final speed of sound map was then constructed as:

(3) $$\begin{array}{c} c_{\mathrm{tissue}}\left(x,z\right)=c_{\mathrm{tissue},\mathrm{global}}M_{\mathrm{background}}\left(x,z\right)\\ +\left(c_{\mathrm{tissue},\mathrm{local}}-c_{\mathrm{tissue},\mathrm{global}}\right)M_{\mathrm{microstructure}}\left(x,z\right). \end{array}$$

The tissue heterogeneity was estimated using an exhaustive search approach, where *µ*_tissue_ values for each of the two microstructural tissue components were systematically varied. Specifically, histology-based phantoms were constructed using different combinations of cell and collagen *µ*_tissue_, selected from a pre-defined range, to simulate in silico ultrasound images. The search range for *µ*_tissue_ was selected to reflect physiologically plausible variations in density and speed of sound based on reported ranges for soft tissues. The in silico images were compared to ex vivo acquired ultrasound data to identify the optimal *µ*_tissue_ configuration. First-order speckle statistics were used to quantify the lowest dissimilarity between regions of interest (ROI) in the in silico and ex vivo ultrasound data, enabling selection of the most representative *µ*_tissue_ values. The ex vivo ultrasound data was acquired on the same tissue as the histology samples were extracted from, imaged in the same plane.

The dissimilarity was quantified using the Jensen-Shannon Divergence (JSD),^32^ defined as:

(4) $$JSD\;(P||Q)\;=\;\frac{1}{2}(\;D_{KL}(P||M)\;+\;D_{KL}(Q||M)),$$

with

(5) $$M\;=\frac{1}{2}\left(P+Q\right),$$

where *P* and *Q* are discrete probability mass functions of the ultrasound envelope data, *M* the mixture distribution of *P* and *Q*, and *D_KL_* the Kullback-Leibler Divergence. The *D_KL_* was defined as:

(6) $$D_{KL}(P||M)=\sum_{x\in X}P\left(x\right)\log_{2}\left(\frac{P\left(x\right)}{M\left(x\right)}\right).$$

The discrete probability mass functions were computed on the normalized ultrasound envelope data using a bin width of 0.03. This bin width was selected based on the Freedman-Diaconis rule, applied to a minimum ROI size of 25 × 25 wavelengths.^33^ The minimum ROI size was determined using in vitro ultrasound data acquired on a scattering phantom exhibiting fully developed speckle. A small convergence study was conducted in which the ROI size was varied, and the Jensen-Shannon divergence (JSD) was monitored to identify the point at which further increases in ROI size no longer affected the divergence values.

The maximum ROI size was constrained by the dimensions of the corresponding histology samples. For the muscle and adipose fat, the largest possible rectangular ROI was used to ensure sufficient statistics. Since the average *µ*_tissue_ was estimated per tissue type, a larger search area was defined in the ex vivo acquired US images. This search area was subdivided into ROIs with 20% overlap, matching the in silico ROI dimensions. The ROIs for the carotid artery and skin were manually delineated. Prior to JSD computation, the ultrasound envelope data was normalized to the 99th percentile.

The histology samples for histology-based phantom construction were divided into two datasets: one to determine *µ*_tissue_ (training set) and one to evaluate the estimated values (test set). Specifically, 11 samples of adipose tissue, 4 carotid artery samples, 9 muscle samples, and 4 skin samples were used to estimate *µ*_tissue_. The evaluation set comprised 11 samples of adipose tissue, 4 carotid artery samples, 4 muscle samples, and 4 skin samples. For both muscle and adipose fat, measurements were performed on two distinctive tissue samples, with a unique local microstructure that influences the ultrasound appearance. To ensure a fair comparison, the derived numerical phantoms were only compared to the specific tissue samples from which the histology samples were taken.

### Isotropic Tissue Phantom

Next to validating the realism of the tissue microstructure the importance of incorporating tissue microstructure was assessed quantitatively. To this end, phantoms were created under the assumption of isotropic normally distributed acoustic properties. These control simulations, lacking any structural features, produce pure, fully developed speckle and serve as a baseline for comparison.

Isotropic scattering was modeled using the speckle intensity approach described by Muller et al.^18^ The magnitude of the speckle intensity was defined by the interquartile range (IQR) of the tissue density distributions in the microstructure-based simulations. The same global acoustic properties listed in Table 1 were used to ensure consistency across simulations.

### Data Collection

For ultrasound acquisition, the same tissue samples were imaged as for the histology with the image planes aligned as precisely as possible. The tissue was submerged in 0.9% physiological salt solution at room temperature (20°C–21°C). Ultrasound images of the adipose fat, carotid artery and muscle were obtained using a Vantage 256 system (Verasonics, Kirkland, WA, USA) equipped with a L11-5v linear array transducer (center frequency 7.6 MHz, pitch 300 µm). In addition, high frequency data were acquired for the carotid artery and skin with the L22-14v linear array transducer (center frequency 18 MHz, pitch 100 µm). Images were acquired using plane wave imaging, steering 41 unfocused plane waves between −20° and +20° for the L11-5v, and −10° to +10° for the L22-14v. The data were beamformed using delay-and-sum beamforming, with a fixed f-number of 3.7, and gridspacing of 50 and 15 µm, for the L11-5v and L22-14v, respectively. A uniform speed of sound of 1500 m/s was used for the adipose fat and carotid artery, for the muscle and skin 1540 m/s was used.

### Simulation Design

Ultrasound simulations were carried out with the open-source k-Wave Toolbox version 1.3^14,15^ in MATLAB version 2023b. The linear array transducers, used in the experiments, were implemented in k-Wave to match the ex vivo imaging experiments as closely as possible. The L11-5v linear array transducer was modeled with 128 elements, with an element width of 270 µm and a pitch of 300 µm. An element width of 80 µm and pitch of 100 µm were used for the simulations mimicking the L22-14v transducer. To decrease staircasing errors resulting from directly sampling the elements to the discrete computational grid, the transducer elements were implemented with an analytical description of the band- limited rectangle function.^18^ The elements were described as a velocity source, transmitting in element’s normal direction. The transmit pulse was defined with a two-way simulated waveform, as provided by Verasonics (Vantage software, version 4.9.2).

The image domain was discretized by defining a uniformly sampled computational grid. For the L11-5v transducer the grid had dimensions of 40 mm × 40 mm, in axial and lateral direction, respectively. The grid spacing corresponded to eight points per wavelength for the center frequency of 7.6 MHz, resulting in 24.7 µm. A computational grid of 15 mm × 30 mm was used for the L22-14v transducer, with a grid spacing of 10.4 µm. A Courant–Friedrichs–Lewy number of 0.3 was used for the time step definition. The same post-processing was applied to the in silico RF channel data as for the experimental data acquired with the Verasonics system. Therefore, the numerical simulations closely mimicked the system’s hardware and image reconstruction.

The transducer implementation was evaluated by comparing the speckle size of a numerical speckle phantom to that of experimental data acquired in a poly(vinyl alcohol) cryogel (PVA) scattering phantom. This physical phantom consisted of 10 wt% PVA and 2 wt% Silicon Carbide particles and is explained in study^34^ in more detail. The speed of sound was estimated using a global autofocusing method, maximizing the speckle power of the reconstructed ultrasound image by varying the reconstruction speed of sound,^35^ resulting in 1504 m/s. An acoustic absorption coefficient of 0.29 dB/(MHz cm) was approximated using a linear fit to the intensity decay of the measured RF channel data. To estimate the speckle size, square-shaped ROIs containing ultrasound envelope data were selected for varying depths, with an axial overlap of 80%. The speckle size was determined by computing the normalized autocorrelation of the envelope data, where the FWHM of the autocorrelation was determined by fitting an ellipse to the central peak of the autocorrelation map. The ellipse fitting was performed using a least-squares approach, without allowing tilt or rotation of the ellipse to ensure axis-aligned measurement in axial and lateral direction. In the simulations the PVA phantom was described with a density of 1000 kg/m^3^, the estimated speed of sound and absorption coefficient, and a speckle intensity of 1 × 10^−8^ (unitless). Square-shaped ROIs with a size of 20 × 20 wavelengths were used, on five ex vivo and five in silico images, resulting in 195 and 230 extracted ROIs for the L11-5v and L22-14v transducers, respectively.

### Ex Vivo Evaluation

An independent set of histology-based phantoms and baseline phantoms were used for evaluation. The difference in first-order speckle statistics was again determined in terms of JSD. Here, ex vivo ROIs were extracted from the search areas with zero overlap. A statistical data analysis was performed to compare the JSD distributions obtained for the histology-based phantom and the baseline method. After testing the JSD for normality using a Lilliefors test, a two-sample Student’s *t*-test or a Mann-Whitney *U*-test was performed to compare the histology-based phantom with the baseline method. For all statistical tests, a *p*-value below .05 was considered to be significant.

The speckle patterns were additionally evaluated with a multi-level texture analysis in terms of the texture anisotropy index (TAI), inspired by the method of Dubois et al.^36^ The TAI was derived from the inertia tensor, which can be interpreted as a local covariance matrix derived from the image gradient.^37^ The inertia tensor was computed on ROIs extracted from B-mode ultrasound data, normalized to the 99th percentile with a dynamic range of 60 dB. For the muscle and adipose fat tissue, a ROI size of 6 mm × 6 mm was used (30 × 30*λ*). For the skin, a ROI size of 3.9 mm × 1.7 mm was used (45 × 20*λ*). Due to the limited speckle content in the carotid artery images, texture analysis could not be reliably performed for this tissue type.

Each ROI was subjected to low-pass filtering using a fourth order Butterworth filter to suppress high- frequency noise while preserving relevant structural information. Multiple cut-off frequencies were applied to enable a multi-level analysis, capturing both fine and coarse texture features. Specifically, cut-off frequencies of (16*λ*)^−1^, (8*λ*)^−1^, (4*λ*)^−1^, and (2*λ*)^−1^ were used, with *λ* denoting the wavelength.

Image gradients (*G*) were calculated using the Sobel operator along the axial and lateral image axis. The gradients were flattened and mean-centered to form the gradient matrix *W_c_*,

(7) $$W_{c}=\left[G_{lateral,i}-{\bar{G}}_{lateral};G_{axial,i}-{\bar{G}}_{axial}\right]$$

to compute the local covariance matrix *C*,

(8) $$C=\frac{1}{N-1}W_{c}^{⊤}W_{c}$$

with *N* the number of pixels in the ROI. The two eigenvalues were retrieved, *λ*_max_ and *λ*_min_, which carry information about the distribution of the gradient within the ROI.^37^ The eigenvalues were used to compute the texture anisotropy index (TAI), defined as:

(9) $$TAI=1-\frac{\lambda_{min}}{\lambda_{max}},$$

where a TAI approaching 1 indicates a strong preferred direction within the ROI, and a TAI approaching 0 indicates isotropy.^36^

### Multi-Layered Tissue Phantom

**Figure 2.**
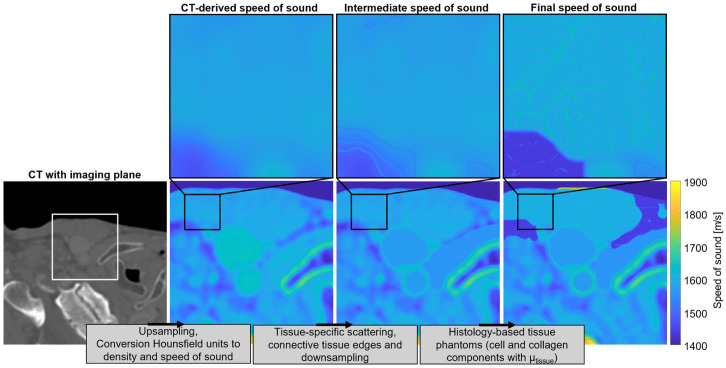
Schematic representation of the workflow to construct the speed of sound map of the multi-layered tissue phantom derived from CT data. Histology-based phantoms of the carotid artery, adipose fat, skin, and muscle were included in the multi-layered tissue phantom. A similar workflow can be followed to obtain the tissue density.

A representative model of the carotid artery region was constructed by integrating the histology-based phantoms into an acoustic medium derived from CT data. An overview of the global steps taken to construct the multi-layered tissue phantom can be found in Figure 2. A single CT frame was selected from the open-source Head and Neck CT Atlas,^38^ and upsampled to a 10 times oversampled grid with a spatial resolution of 2.47 µm. The CT frame was converted to a spatial density map using multiple linear fits based on the experimental data given by Schneider et al.,^39^ implemented in the k-Wave toolbox. Corresponding speed of sound values were approximated by the empirical relationship found by Mast.^31^ For tissues other than adipose fat, carotid artery, muscle or skin, tissue-specific speckle intensity, and absorption coefficients were assigned based on density intervals, following the methodology of a previously developed CT-based simulator.^20^ The speckle intensities were defined a factor 50 higher to match the intensity achieved with the histology-based phantoms. To enhance realism, transitional edges between regions of differing speckle power were defined using a Sobel edge detection. These edges had a thickness of 1.23 µm. Acoustic properties of connective tissue were assigned to the edges, with a density of 1026 kg/m^3^, speed of sound of 1545 m/s, and absorption coefficient of 1.17 dB/(MHz cm).^30^ Additionally, the thyroid was manually delineated and added to the CT-based phantom, after smoothing the segmentation using a Gaussian filter (*σ* = 2.96 µm) with a density of 1050 kg/m^3^, speed of sound of 1500 m/s, absorption coefficient of 1.2 dB/(MHz cm) and speckle intensity of 5 × 10^−7^ (unitless).^30^ The CT-based phantom was subsequently low-pass filtered and downsampled to match the computational grid.

The histology-based phantoms were embedded in the CT-derived acoustic medium. The value of *µ*_tissue_ was scaled to achieve realistic contrast between tissues, with a factor 2 smaller for adipose fat and muscle. As the in vivo carotid artery is exposed to intraluminal pressure, the spatial microstructure maps derived from histology were warped to have a circular shape to enhance realism. An inner radius of 2.5 mm was used, as measured in the CT data. It should be noted that there was no correction for arterial wall thickness with respect to the inner radius. The histological muscle microstructure did not cover the entire muscle area in the CT-based medium. To enable full coverage, the muscle microstructure was extended using the clone stamp tool in Adobe Photoshop (version 26.9). Additionally, the muscle phantom was rescaled such that the muscle fascicles showed a texture visually matching the appearance of the neck muscle, since there might be a difference in fascicle diameter between the porcine leg muscle used in the original phantom and neck muscles, as these are highly varying in literature.^40,41^ Ultrasound was simulated for the L11-5v transducer, with the same acquisition process previously described. Baseline phantoms were also embedded in the CT-based medium for comparative analysis.

## Results

### Transducer Validation

**Figure 3.**
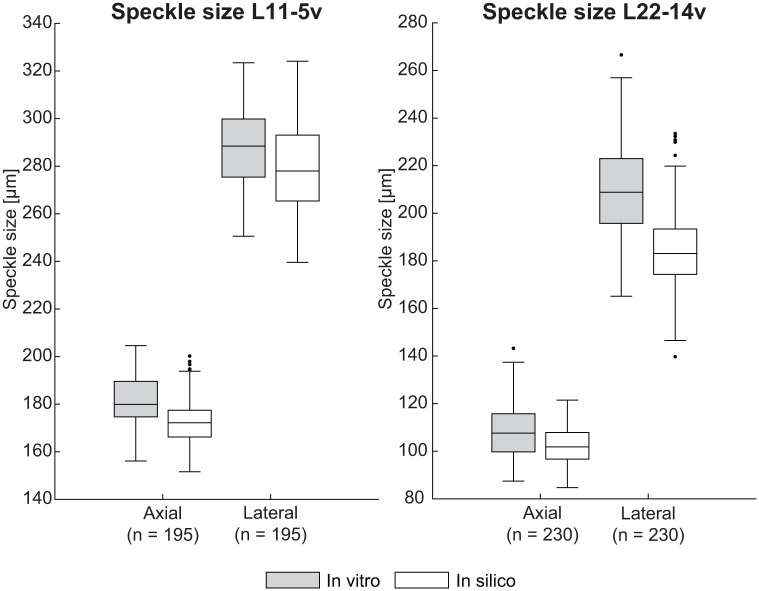
Speckle size distributions for the L11-5v and L22-14v linear array transducers acquired on a poly(vinyl alcohol) phantom.

The transducers and hardware implementation were evaluated by analyzing the axial and lateral speckle sizes at various depths. Figure 3 presents the distributions of axial and lateral speckle sizes for the L11-5v and L22-14v transducers. For the L11-5v transducer, the axial speckle sizes measured in vitro and in silico are comparable, with values of 180 ± 15 µm and 172 ± 11 µm (median ± IQR). The lateral speckle size only differs 10 µm between in vitro and in silico measurements, with values of 288 ± 24 µm and 278 ± 28 µm, respectively. For the L22-14v transducer, the axial speckle size is also similar, with in vitro and in silico values of 108 ± 16 µm and 102 ± 11 µm, respectively. A larger difference of 26 µm can be observed for the lateral speckle size, 209 ± 27 µm (in vitro) and 183 ± 19 µm (in silico).

### Histology-Based Tissue Phantom

**Figure 4.**
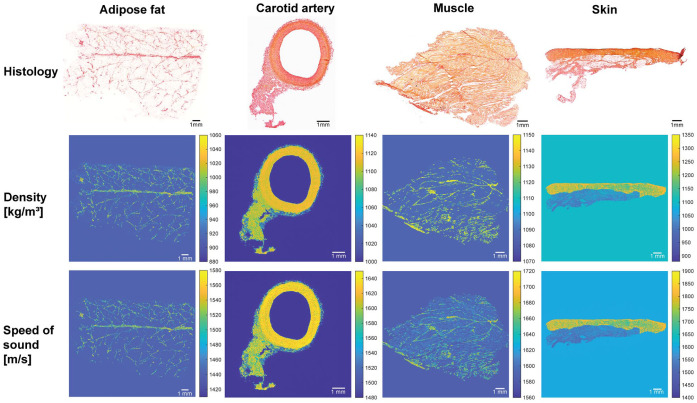
Histology images of all four tissue types with the resulting histology-based phantom for ultrasound simulation. Top row: histology stained with picrosirius red staining. The middle and bottom row show the numerical phantom for ultrasound simulation, with the density in [kg/m^3^] and the speed of sound in [m/s].

Local tissue heterogeneity was estimated for adipose tissue, carotid artery, muscle, and skin, with values summarized in Table 2. As expected, collagen shows a higher *µ*_tissue_ than cytoplasm. These values were used to generate the histology-based phantoms in Figure 4. The resulting density and speed of sound range between 900 and 1350 kg/m^3^ and 1440–1900 m/s, respectively. These are of the same magnitude as one would expect for biological tissue. Picrosirius red staining clearly distinguishes collagen (red) from cytoplasm (yellow), as shown in the top row of Figure 4. Distinct structural features can be observed for each tissue type. Adipose tissue shows fascia lines and well-defined collagen structures surrounding the fat cells. The carotid artery displays the intima, media, and adventitia layers. Skeletal muscle was shown in mainly longitudinal direction, where the fascicles were surrounded with collagen-rich perimysium.

**Table 2.** Estimated local acoustic heterogeneity (*µ*_tissue_) values and baseline speckle intensity. The first two columns list the local acoustic heterogeneity, derived for the collagen and cytoplasm components per tissue type. In the third column the speckle intensity used in the baseline simulations is reported.

Tissue	Collagen *µ*_tissue_	Cytoplasm *µ*_tissue_	Speckle intensity
Adipose fat	1.14	1.035	2.49 × 10^−8^
Carotid artery	1.05	1.035	2.70 × 10^−8^
Skeletal muscle	1.07	1.005	4.73 × 10^−8^
Skin	1.15	1.020	3.40 × 10^−7^

**Figure 5.**
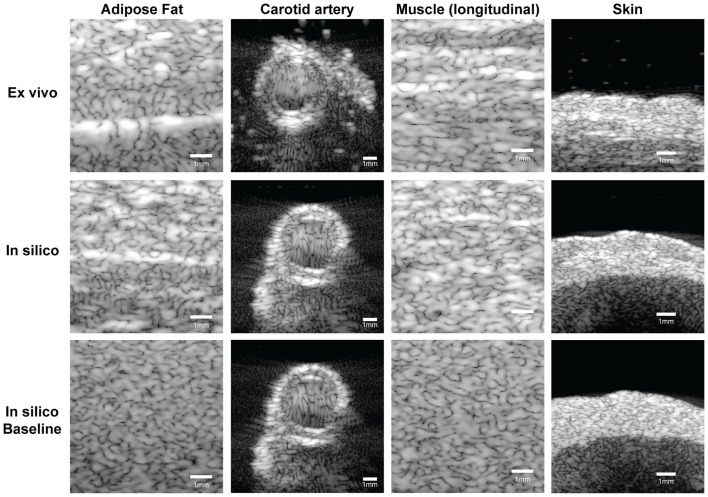
B-mode ultrasound images of all four tissue types. The depicted dynamic range is 60 dB. Top row: speckle patterns obtained from ex vivo animal tissue; middle row: images acquired on the histology-based phantom, and bottom row: the baseline phantom for comparison. The first three columns (adipose fat, carotid artery, and muscle) show ultrasound data acquired with the L11-5v transducer, in the fourth column (skin) the L22-14v transducer was used.

Figure 5 presents a qualitative comparison of B-mode ultrasound images simulated with the histology- based phantom and baseline phantom to the ex vivo acquired data. The histology-based simulations exhibit higher realism compared to the baseline. The adipose fat simulation clearly shows the fascia line, closely resembling the ex vivo appearance. A comparable carotid artery structure was found for both methods, with similar shadowing artifacts and axial lobes, consistent with the ex vivo data. The muscle tissue simulation shows a similar pattern as the ex vivo data. In the skin tissue, the layered structure is well preserved. Multiple layers can be distinguished, reflecting the dermis and hypodermis. The speckle intensity, used in the baseline method, is listed in Table 2.

**Figure 6.**
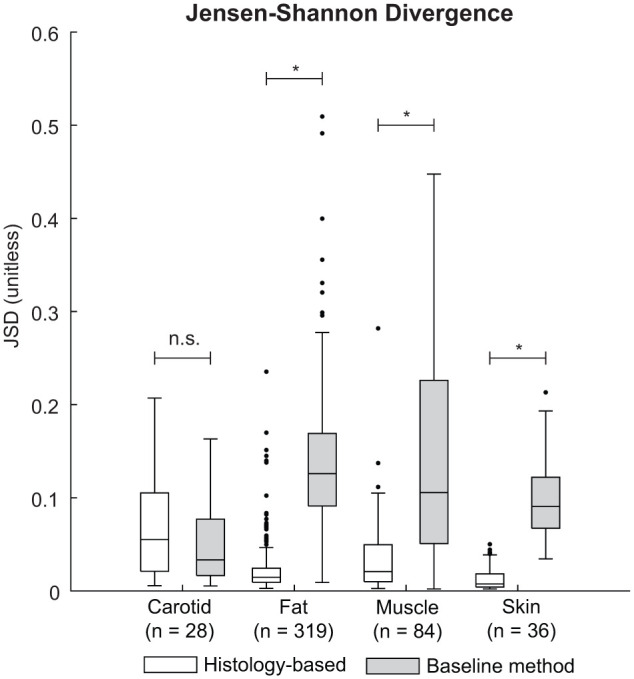
Summary statistics of the Jensen-Shannon Divergence (JSD) obtained for the histology-based tissue phantom (white) and the baseline phantom (gray). Significant differences between the distributions are indicated with asterisks. n.s = not significant. *p ≤ .001.

To quantify the dissimilarity between the in silico and ex vivo speckle patterns, the JSD was computed for each tissue type. Summary statistics are shown in Figure 6. The histology-based phantom yielded low JSD values for the adipose fat (0.015), muscle (0.021) and skin (0.007), compared to the baseline, (0.126, 0.106, and 0.091, respectively). These observations indicate a close match to the ex vivo first-order speckle statistics. The Lilliefors test revealed a normal distribution for the skin tissue simulated with the baseline phantom (*p* = .12), for all other JSD distributions no normal distributions were found (*p*-values ranging between *p* ≤ .001 and *p* = .011). Therefore, a Mann-Whitney *U*-test was used to test for equal medians. Significant differences were found between the histology-based and baseline phantoms for the adipose fat, muscle, and skin tissue, with *p* ≤ .001 for all tissues, respectively. For the carotid artery, no significant difference between the two phantoms was found (*p* = .14), with a median JSD value for the histology-based phantom of 0.055 compared to the baseline of 0.033.

**Figure 7.**
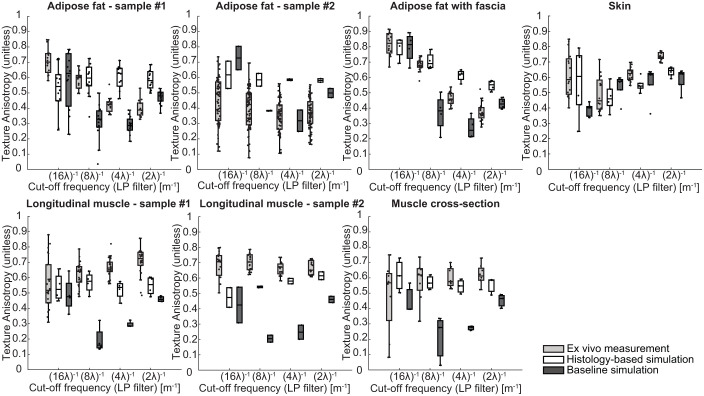
Overview of the texture analysis across multiple tissue types and orientations. Each subplot shows the texture anisotropy index (TAI) as a function of low-pass filter cut-off frequency [m^−1^], comparing ex vivo data (light gray), the proposed microstructure tissue phantom (white), and the baseline method (dark gray). The analysis was performed on seven distinct tissue configurations: longitudinal muscle (two samples), muscle cross-section, adipose fat (two samples), adipose fat with thicker fascia, and skin.

The texture analysis further supports the high similarity, as illustrated in Figure 7, which presents the TAI per tissue type across four spatial cut-off frequencies. As the analysis was conducted for each tissue in its corresponding imaging plane, seven distinct evaluations are shown: two longitudinal muscle samples, muscle cross-section, two adipose fat samples without fascia, adipose fat with fascia, and skin. Overall, the histology-based simulations demonstrate a closer match to the ex vivo measured ultrasound data compared to the baseline simulations. Specifically, adipose fat sample 1 aligns well with the measured data at (8*λ*)^−1^, although the baseline phantom seems to outperform the histology-based phantom across different spatial cut-off frequencies. A difference in ex vivo median TAI values of 0.21 and 0.18 was observed for (16*λ*)^−1^ and (8*λ*)^−1^, respectively, between the two adipose fat samples. In addition, adipose fat sample 2 exhibited a wide range of TAI values, likely due to its large size and high heterogeneity. For adipose fat with large fascia in the imaging plane, a strong match is observed at lower cut-off frequencies, particularly at (8*λ*)^−1^ (difference of 0.002 vs. 0.27 for the baseline), though the histology-based phantom shows a higher anisotropy at higher cut-off frequencies.

Both longitudinal muscle samples show strong agreement with the measured data, particularly at lower spatial frequencies ((16*λ*)^−1^ and (8*λ*)^−1^), where sample 1 exhibits minimal differences in median TAI values (−0.005 and 0.064) versus substantially larger discrepancies in the baseline (0.04 and 0.48). The reported differences represent the differences between the median TAI compared to the ex vivo measured ultrasound data. Similarly, the histology-based muscle cross-section displays high resemblance across all frequencies, with an average median difference of 0.04, markedly lower than the 0.24 observed for the baseline. The skin samples consistently outperform the baseline across all frequencies, with notably smaller differences at lower cut-off frequencies (−0.02 and −0.01 for (16*λ*)^−1^ and (8*λ*)^−1^, respectively) compared to the baseline (0.18 and −0.12), and comparable or improved performance at higher frequencies.

### Multi-Layered Tissue Phantom

**Figure 8.**
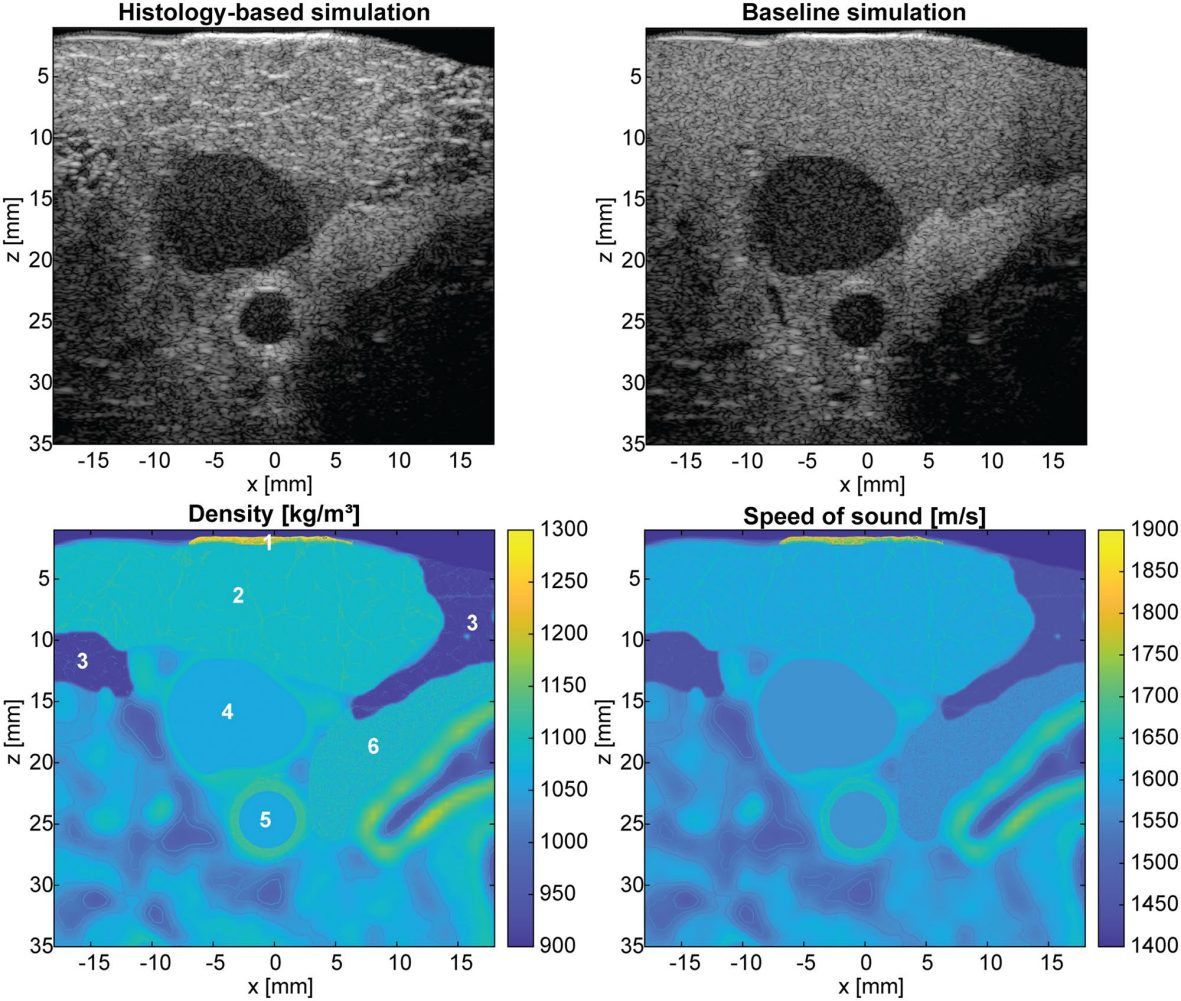
Simulation of the carotid artery (neck region). Top left: histology-based ultrasound simulation combining skin, adipose fat, muscle and carotid artery with CT-based geometry. Top right: baseline simulation. Bottom row: density and speed of sound map combined with CT-based geometry. Different tissues of interest are labeled in the density map: skin (1), skeletal muscle (2), adipose fat (3), jugular vein (4), carotid artery (5), and thyroid (6).

A simulation of the carotid artery region is shown in Figure 8. In the top row, ultrasound simulations using the histology-based phantoms and the baseline phantoms are presented. It can be clearly observed that the histology-based phantom adds image realism compared to the baseline phantom. The fascia lines of the adipose fat are clearly visible, with the homogeneous thyroid region below. The muscle cross-section adds texture, which is visible in the upper imaging region above the jugular vein. The carotid artery is circular and with a closer look the media and adventitia layer can be distinguished. In the bottom two subfigures the spatial density and speed of sound distributions of the tissues are depicted.

## Discussion

This study presents a method to construct histology-based microstructural tissue phantoms for realistic ultrasound data simulation. The proposed method demonstrates a variety of tissues, based on the spatial organization of cells and collagen fibers, showing a significant improvement in the simulation of tissue-specific speckle patterns.

Scattering properties depend significantly on the assigned local acoustic properties. In this research, we estimated the relative acoustic heterogeneity *µ*_tissue_ on a micrometer scale for the collagen and cytoplasm components. It should be emphasized that *µ*_tissue_ does not necessarily correspond to actual physical quantities such as local density and speed of sound. Nevertheless, the estimated ratio between cytoplasm and collagen components is still effectively used to simulate realistic ultrasound images, under the assumption of linear wave propagation. Acoustic microscopy could be considered to further verify the accuracy of the estimation approach.

To estimate the performance of the histology-based phantom, a comparison was made with a baseline phantom assuming isotropic scattering. Overall, the histology-based phantom shows a higher similarity to the measured ultrasound data compared to the baseline. For the adipose fat, muscle and skin, an average improvement by a factor of 8 was observed in terms of JSD. Compared to a previous CT-based simulation of IVUS data,^20^ the proposed numerical phantom shows similar performance for the carotid artery. Specifically, the IVUS simulator achieved a JSD of 0.066 for the arterial wall, while our numerical phantom achieved a JSD value of 0.055. This slight improvement could be explained by the dominance of specular reflections and the thin vessel wall.

For the texture analysis, we focused specifically on the TAI as a metric, which provides a simple and interpretable measure of directional alignment, making it particularly suitable for tissues like muscle and fat.^36^ Unlike commonly used texture descriptors such as the gray-level co-occurrence matrix or local binary patterns, which yield complex or less intuitive features, TAI offers a single value metric that is rotational-invariant and shown to be robust for varying imaging settings.^36,42^

A texture analysis was performed on adipose fat, muscle and skin using the TAI across multiple spatial cut-off frequencies to characterize both coarse and fine structural features. Notably, Figure 7 reveals distinctive TAI profiles over frequency for different tissue types. For the adipose fat, the TAI also varied between tissue sample 1 and 2, and a high variance can be observed within the measurements in sample 2. These findings highlight the importance of using matched tissue pairs between simulation and experiment and ensuring histology samples are acquired from the same anatomical region to enable meaningful comparisons. Looking closer at Figure 7, a high agreement in texture was found for the muscle and skin compared to the baseline method across all spatial cut-off frequencies. In contrast, the adipose fat tissue demonstrated discrepancies in TAI for spatial frequencies of (4*λ*)^−1^ and (2*λ*)^−1^, indicating subtle differences in speckle appearance between ex vivo and insilico, which can be seen in Figure 4. The in silico speckle appears to be smaller, which may be attributed to the lack of the elevational direction in the simulations and/or to variations of the assumed speed of sound, since this value was not estimated for the tissue but extracted from literature.

The appearance and intensity of the ultrasound speckle pattern is highly dependent on the number of scatterers per resolution cell.^43^ It is therefore important to accurately model the ultrasound transducers in terms of spatial resolution. In vitro measurements on a PVA scattering phantom are used to accurately model the probe resolution. For the L11-5v transducer, a difference in axial and lateral speckle size of only 8 and 10 µm, respectively was found, which is very small (4%–5%) compared to the ultrasound wavelength. These results indicate that the transducer was modeled with sufficient accuracy to simulate realistic ultrasound speckle patterns. The L22-14v resulted in an axial speckle size difference of 6 µm, which is only 7% compared to the wavelength. A larger lateral difference of 26 µm was observed, which could be attributed to the fact that the simulations were carried out in two dimensions, disregarding the elevational spread of the ultrasound beam. This is one of the main limitations of this study. Future work should investigate the influence of the elevational direction in the resulting ultrasound speckle pattern, possibly further enhancing image realism, albeit with increased computational demands.

The ability to simulate realistic ultrasound images based on histological microstructure rises new opportunities for training, validation, and device development. These phantoms can serve as ground truth for image analysis techniques, such as strain imaging or segmentation algorithms, where in vivo validation is challenging.^5,6^ Furthermore, these simulations offer a controlled environment for studying the sensitivity and robustness of image acquisition or processing techniques to variations in tissue morphology, imaging settings, or device parameters.^44^ Realistic simulations can be achieved by combining the histology-based phantoms with patient geometries derived from CT. An example of an imaging scene of the carotid artery is shown in Figure 8. Here, one can appreciate the added texture and realism the histology-based phantom brings compared to the simulation using the baseline phantom. The use of histology samples enables detailed anatomical realism, however, it is inherently time-consuming and not scalable. Additionally, prior information from histology is not always available in practice. Future work will focus on computationally generating synthetic tissue microstructure based on average cell sizes and orientation. This allows to automate and scale up phantom creation, further broadening the applicability of this simulation framework.

## Conclusion

This study introduces a novel framework for constructing histology-based tissue phantoms that significantly enhance the realism of ultrasound simulations. By incorporating tissue heterogeneity derived from whole-slide histology images, including collagen fiber orientation and cell anisotropy, the proposed method enables the generation of ultrasound images that closely resemble ex vivo measurements. Quantitative validation using JSD and TAI confirms that the simulated speckle patterns improved compared to baseline isotropic phantoms.

The integration of the histology-based phantoms with CT-based anatomical geometries further demonstrates the potential for realistic in vivo scene simulation. While the current approach relies on histology data, future work will focus on synthetic microstructure generation to support scalable dataset creation. This advancement opens new avenues for training, validation, and development of ultrasound imaging technologies, offering a robust platform for developing and evaluating image analysis algorithms strategies under controlled and anatomically realistic conditions.

## Appendix A: Details on Tissue Segmentation Parameters

Tissue microstructure was assessed from the histology images by means of a segmentation. To ensure correct decomposition into a cell and collagen component, the images were segmented into multiple clusters which were later merged. The number of clusters and morphological operations were optimized for per tissue type. An overview of the parameters used is given in Table 3 below.

**Table 3.** Details on tissue segmentation.

Tissue type	Pixel spacing	*k*-Means clusters	Components	Post-processing
Adipose fat	1.30 µm	6	Cell walls and collagen	Morphological closing of cell walls and collagen, SE 2 pixels.
				Structures with an area < 1200 pixels were removed.
Carotid artery	0.87 µm	9	Smooth muscle cells and collagen	Smooth muscle cells: morphological closing followed by opening.
				SE 1 pixel. Collagen: closing followed by opening, SE of 2 pixels.
				Structures with an area < 120 pixels were removed.
Muscle	1.73 µm	9	Myocytes and collagen	Morphological opening of myocytes with SE of 3 pixels.
				For the collagen a morphological closing followed by a dilation
				with SE of 3 pixels, structures < 1000 pixels were removed.
Skin	1.73 µm	7	Epidermis, collagen-rich tissue and skin cells.	Morphological closing with SE of 2 pixels.
				Structures < 50 pixels were removed from the epidermis,
				from the collagen structures < 20 pixels.
